# Research on the Influence Law and Mechanism of Regenerated Ceramic Tile Form and Replacement Rate on the Mechanical Properties of Ultra-High-Performance Concrete

**DOI:** 10.3390/ma18133028

**Published:** 2025-06-26

**Authors:** Xiuying Yang, Yiwu Xing, Zhen Wang, Shixin Duan, Guodong Zhao, Jie Song, Zhaohui Xiao

**Affiliations:** 1 School of Architecture and Engineering, Liaocheng University, Liaocheng 252000, China; 2 School of Civil Engineering, Shandong Jiaotong University, Jinan 250357, China; 3 Shandong Quality Inspection and Testing Center of Construction Engineering Co., Ltd., Jinan 250108, China; 4 Shandong Academy of Building Research Co., Ltd., Jinan 250108, China

**Keywords:** ultra-high-performance concrete, regenerated ceramic waste, mechanical properties, microstructure analysis

## Abstract

Ultra-high-performance concrete (UHPC) has gained widespread application across various domains owing to its superior properties. Nevertheless, the high cement content and associated costs present challenges, including significant shrinkage of the cement matrix and economic considerations. Using industrial by-products or waste to replace some raw materials is one of the effective solutions. Meanwhile, China’s ceramic industry generates a large amount of waste every year. Applying ceramics in UHPC can effectively solve these problems. This study explores the use of recycled tile waste as a sustainable alternative to reduce the use of natural aggregates and cement and enhance the performance of UHPC. To investigate the impact of recycled ceramics on the mechanical properties of UHPC, three preparation methods were employed: (1) single incorporation of ceramic tile aggregate (CTA) to replace fine aggregates (0–100%), (2) single incorporation of ceramic tile powder (CTP) to replace cementitious materials (0–20%), and (3) dual incorporation of both CTA and CTP. The effects of different preparation methods and substitution rates on mechanical properties were evaluated through compressive and flexural strength tests, and microstructure analyses were conducted by scanning electron microscopy (SEM) and mercury intrusion porosimetry (MIP). The test results show that the compressive strength and flexural strength of UHPC increased with an increase in the ceramic particle substitution rate and reached the maximum value at a 100% substitution rate. On the contrary, ceramic powder substitution initially reduced the compressive strength, and it slightly recovered at a substitution rate of 10%. However, the bending strength decreased with an increase in the substitution rate of the ceramic powder. When ceramic particles and ceramic powder were used in combination, the compressive strength was the highest when 100% ceramic particles and 20% ceramic powder were used as substitutes. The maximum flexural strength occurred when 100% ceramic particles or 5% ceramic powder was used as a substitute. This study demonstrates that recycled ceramic waste can effectively enhance the mechanical properties of UHPC, providing a sustainable solution for reducing cement consumption and improving the performance of concrete.

## 1. Introduction

In recent years, China has maintained its position as the world’s largest producer of building ceramics. From 2012 to 2022, China’s annual output ranged from 8.1 billion to 8.6 billion square meters, accounting for more than half of the global output [1]. Ceramic waste is increasing with the growth in people’s daily use demand [2], and the treatment of waste ceramics has also drawn attention. Due to the high treatment cost of waste ceramics, a simple landfill treatment is usually implemented. However, due to the difficulty in degrading ceramic waste, improperly handled ceramic waste can have adverse effects on the soil and groundwater [3,4]. UHPC has been widely applied in various fields due to its excellent performance. In order to achieve an excellent performance, up to 1200 kg of cement is required per cubic meter of concrete [5]. In the process of cement production, large amounts of CO_2_ are produced. It is estimated that over the next 33 years, the consumption of cement in concrete manufacturing may emit an additional 85–105 Ct CO_2_ [6]. In addition, refined quartz sand is usually selected as an aggregate when making UHPC. At present, the over-exploitation of natural sand and gravel resources in China has led to shortages of resources in some areas and also has a negative impact on the ecological environment [7]. Not only China but also the rest of the world is facing a shortage of natural aggregate resources. Therefore, it is necessary to find suitable substitutes to replace natural aggregates [8].

Compared with natural aggregates, aggregates made from recycled waste ceramics have good physical properties. They have increased voids and greater crushing, impact, and abrasion values compared with natural crushed stone [9]. In addition, SiO_2_ and Al_2_O_3_ account for large proportions of the chemical compositions of waste ceramics. The content of SiO_2_ is between 61.72% and 78.62%, and the content of Al_2_O_3_ is between 10.56% and 21.31% [10,11,12,13]. This also gives waste ceramics potential pozzolanic activity. These characteristics of waste ceramics meet the requirements for their use as admixtures or aggregates in concrete production.

In recent years, in order to solve the above problems, many scholars have recycled waste ceramics and incorporated them into concrete production and applications. Waste ceramics mixed into concrete can be used as aggregates or partially recycled as a substitute for cement. When replacing natural aggregates, Sivakumar et al. [14] found that when ceramic tiles were used as a substitute for fine aggregates in concrete, they had a positive effect on the compressive strength and flexural strength of the concrete, which is attributed to the filling effect provided by the ceramic aggregates. Zhang et al. [15] also found that recycled ceramics used as aggregates can improve the mechanical properties of the concrete. Under the condition of a 100% replacement rate, the compressive strength and flexural strength of the concrete increased by 15.5% and 26.5%, respectively. In addition, Nepomuceno et al. [16] studied the influence of different replacement rates of ceramic aggregates on the compressive strength, flexural strength, and splitting tensile strength of concrete through design experiments. The results showed that the mechanical properties of concrete decreased to varying degrees with the increase in the aggregate replacement rate. Zareei et al. [17] conducted an experimental study on the mechanical properties of high-strength concrete mixed with recycled ceramic aggregate. Under the conditions of 20%, 40%, and 60% replacement rates, it was found that the recycled ceramic concrete with a 40% replacement rate had the best mechanical properties. When grinding powder to replace a part of cement, Chen et al. [18] used SEM and EDS to detect the hydration products and microstructure of ceramic powder, which confirmed that the addition of ceramic powder had a positive effect on the microstructure of the concrete. Xu et al. [19] showed that replacing cement with ceramic waste powder at a mass fraction of 35% can reduce the width of the concrete interface transition zone, the overall porosity and the number of mesopores by 15.7%, 4.4% and 42.0%, respectively. Li et al. [20] discussed the influence of the addition of ceramic powder on the mechanical properties of concrete. The test results showed that the compressive strength and tensile strength of mortar decreased with the increase in the replacement rate of ceramic powder replacing cement. In addition, when the content of ceramic powder is greater than 20%, the strength of concrete will further decrease [21].

To sum up, although UHPC has excellent mechanical properties, its high use of cement will greatly increase the cost and energy consumption, and its use of quartz sand as aggregate will also increase the cost. At present, there is a shortage of natural sand and stone resources, which is also one of the reasons restricting the development of UHPC. The recycling of waste ceramics is mainly used in ordinary concrete and high-performance concrete, and the application of waste ceramics in UHPC needs further research [15].

Therefore, to solve the above problems, this study designed three additional methods of ceramics in UHPC: the use of single ceramic particles to replace aggregates (with substitution rates of 0%, 20%, 40%, 60%, 80%, and 100%), single ceramic powder to replace cement (with substitution rates of 0%, 5%, 10%, 15%, and 20%), as well as double ceramic particles and ceramic powder were studied. The compressive test and bending test were designed to investigate the impact of various addition methods and dosages on the mechanical properties of UHPC. The microstructure and porosity of UHPC mixed with recycled ceramic tiles were examined using SEM and MIP tests. According to this idea, this study is arranged as follows. In Section 2, the raw materials, test instruments, and test methods are described. In Section 3, the experimental results of compressive strength test, flexural strength test, SEM, and MIP are described in detail, and the influence of waste ceramics on UHPC is discussed. In Section 4, the conclusions of this study and the prospects for future work are given.

## 2. Experiment

### 2.1. Raw Materials

The primary materials used in the experiment include P·O52.5 cement, silica fume, tile particles, tile powder, quartz sand, steel fiber, water reducer, and water.

The waste tiles used in the test are the tile waste materials that result from being cut and discarded by the tile manufacturer. Before the test, the surface of the waste tiles should be thoroughly cleaned to ensure that there are no other obvious impurities present. When dealing with discarded tiles, the initial manual crushing is carried out with a hammer first. Then, a jaw crusher is used for secondary crushing of the tiles. The crushed tiles are placed in a vibrating screen machine to separate the tile particles, which have a particle size range of 0.15 mm to 2.36 mm.

**Figure 1:**
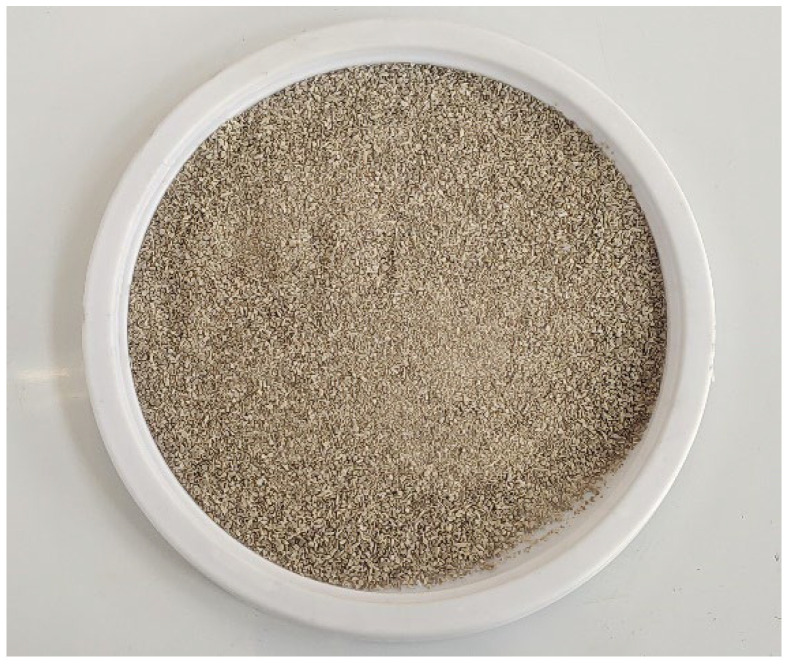
CTA.

**Figure 2:**
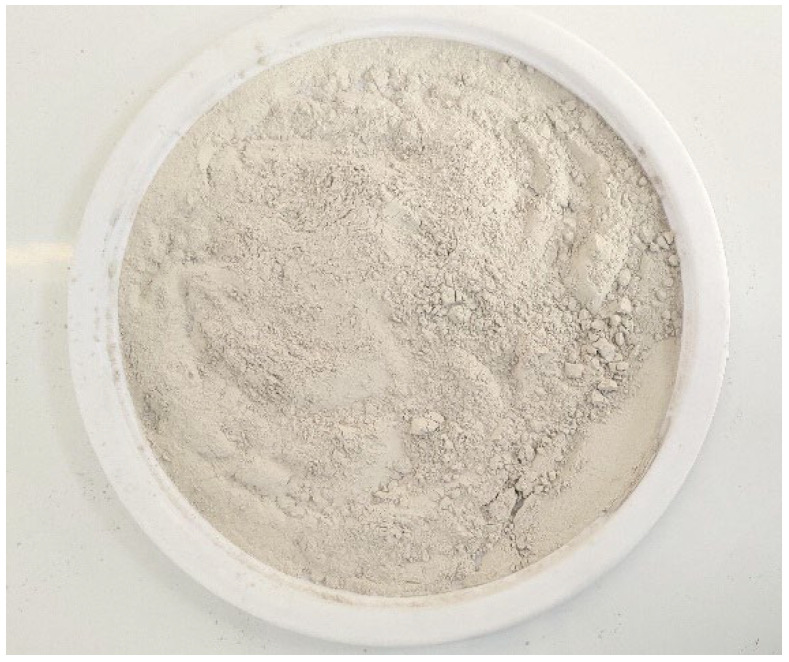
CTP.

The cement used in the test was P·O52.5 ordinary Portland cement produced by Weifang Yangchun Cement Company in Weifang, China, with a specific surface area of 381 m^2^/kg. The chemical composition of the cement is shown in Table 1. Silica fume is a high-performance silica fume produced by Sichuan Langtian Company in Chengdu, China, with a SiO_2_ content of 96.52% and a specific surface area of 2.37 × 104 m^2^/kg. The CTA is obtained by crushing the waste tiles with a jaw crusher and then screening them, as shown in Figure 1. Its Mohs hardness is 6. The gradation of the CTA is shown in Table 2. According to the requirements of the “Sand for Construction” (GB/T 14684-2022) [22] specification, the material properties of recycled aggregates from waste ceramic tiles are shown in Table 3. As shown in Figure 2, CTP is obtained by crushing tile particles with a jaw crusher and then further grinding them in a ball mill. The SiO_2_ content of quartz sand is 99.96%, and its Mohs hardness is 7. Quartz sand with a particle size of 0.15 to 2.36 mm is selected for mixed use. The length of the steel fiber is 10 to 20 mm, the diameter is 0.10 to 0.25 mm, and the tensile strength is not less than 2850 MPa. The water-reducing agent is a high-efficiency polycarboxylate water-reducing agent (pure water-reducing type), with a water reduction rate of over 65% and a solid content of 39.6%.

### 2.2. Specimen Design and Production

The water–binder ratio of UHPC is generally lower than that of ordinary concrete, ranging from 0.14 to 0.2. In the experiment of this paper, the water–binder ratio selected is 0.18. The cementitious materials include cement, silica fume, and tile powder, which are used as substitutes for cement. Among them, the dosage of silica fume is 10% of the cementitious materials, and the proportions of tile powder replacing cement are 5%, 10%, 15%, and 20%, respectively. The cement–sand ratio used in the experiment was 1.0; the dosage of the water-reducing agent was 1.5% of the mass of the cementitious material, and the dosage of steel fiber was 2% of the concrete volume. The basic mix ratios are shown in Table 4. The basic quantities of each material are shown in Table 5.

In this experiment, the proportion of waste tile particles replacing natural quartz sand aggregates was six groups (0%, 20%, 40%, 60%, 80%, 100%), and the proportion of tile powder replacing cement was five groups (0%, 5%, 10%, 15%, 20%). A total of 30 specimen groups were designed, consisting of cubic specimens (100 mm × 100 mm × 100 mm) for compressive strength testing and prismatic specimens (100 mm × 100 mm × 400 mm) for flexural testing. Each group consisted of six cubic specimens for compression tests and three prismatic specimens for flexural tests, resulting in a total of 270 specimens. 

Put the tile aggregates, tile powder, cement, quartz sand, and silica fume into the single-shaft horizontal concrete mixer and pre-stir for 1 min to ensure even distribution. Then, mix the water-reducing agent with water evenly and add it to the mixer, stirring for 3 to 5 min until the concrete becomes fluid. Slowly add the steel fibers and continue to stir for 1 min. After that, it can be loaded into the mold for vibration and smoothing, and then proceed to the next step of curing. The fabricated specimens were cured under the moist curing conditions prescribed by the Chinese National Standard GB/T 50081-2019 [23].

### 2.3. Mechanical Property Test

#### 2.3.1. Compressive Strength Test

**Figure 3:**
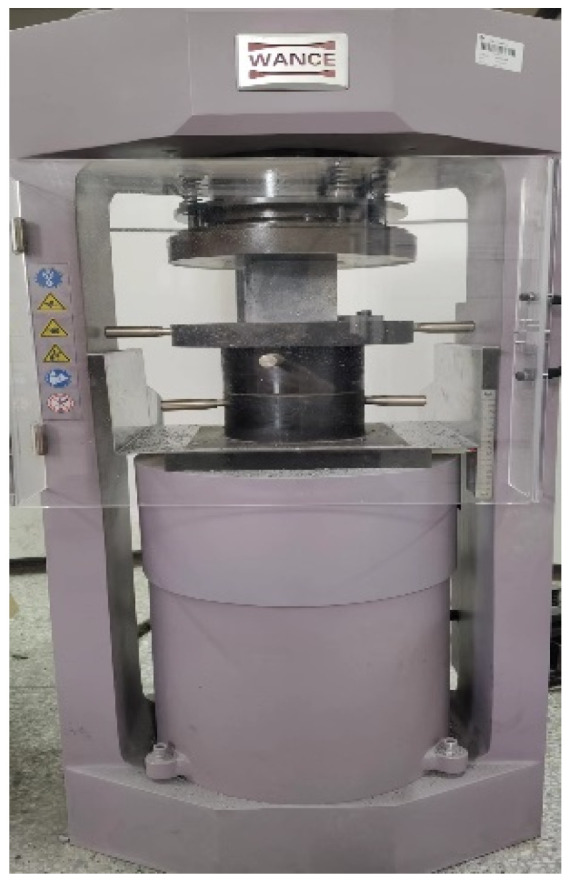
Compressive strength.

The cubic compressive strength of UHPC at 28 days of age was tested using the HCT206A 2000 KN hydraulic servo pressure testing machine from Wance Company in Shenzhen, China, as shown in Figure 3. A total of 20 groups of specimens were poured into the cases of single and double admixtures. The cubic compressive strength test was determined in accordance with the “Standard Test Method for Ultra-High Performance Concrete” (T/CECS 864-2021) [24]. The loading rate is selected as 1.2 MPa/s, and the specimen size is 100 × 100 × 100 mm.

The cubic compressive strength value of UHPC should be calculated according to Equation (1).

(1) $$f_{cu}=\frac{F}{A}$$

In the formula: $f_{cu}$—compressive strength of concrete cubic specimens (MPa). The calculation result should be accurate to 0.1 MPa.

*F*—specimen failure load (N).
*A*—the bearing area of the specimen (mm).

Meanwhile, the determination of the cubic compressive strength value of UHPC specimens should meet the following conditions. Take the average value of the cubic compressive strength of the specimens as the compressive strength value of this group of specimens, accurate to 0.1 MPa. When the difference between a particular test value and the average value exceeds 10% of the average value, it should be discarded, and the average value of the remaining test values should be taken as the compressive strength value of this group of specimens.

#### 2.3.2. Flexural Strength Test

**Figure 4:**
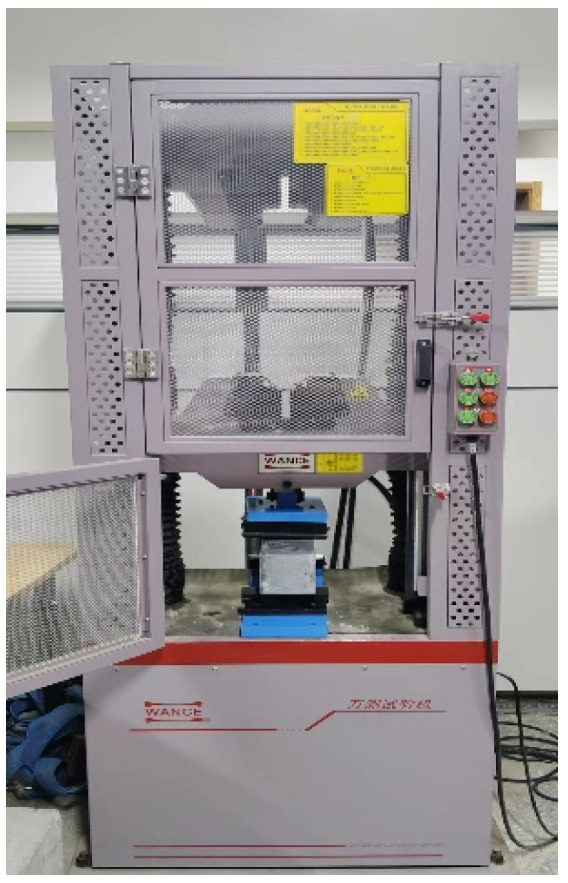
Flexural strength.

The bending strength of 28-day-old UHPC was tested using the HUT305A 300 KN hydraulic servo pressure testing machine of Wance Company in Shenzhen, China, as shown in Figure 4. A total of 20 groups of specimens were poured in both single-admixture and double-admixture cases. The flexural strength test of concrete was determined in accordance with the “Standard Test Method for Ultra-High Performance Concrete” (T/CECS 864-2021) [24]. The loading rate was selected as 0.12 MPa/s, and the specimen size was 100 × 100 × 400 mm.

The flexural strength value of UHPC should be calculated according to Equation (2).

(2) $$f_{w}=\frac{F_{\max}L}{bh^{2}}$$

In the formula: $f_{w}$—flexural strength of concrete cube specimens (MPa). The calculation result should be accurate to 0.1 MPa.

*F_max_*—the maximum load during the bending test process (N).
*L*—the span between the supports of the test beam (mm).
*b*—the width of the cross-section of the specimen (mm).
*h*—the height of the cross-section of the specimen (mm).

The flexural strength of UHPC specimens was determined according to the following protocol. The mean flexural strength of specimens within a test group, calculated to a precision of 0.1 MPa, was adopted as the representative flexural strength value for that group. When either the maximum or minimum flexural strength value within a group deviated by more than 15% from the median value, those outliers were excluded. The flexural strength of the group was then calculated as the mean of the remaining valid specimens.

### 2.4. SEM

**Figure 5:**
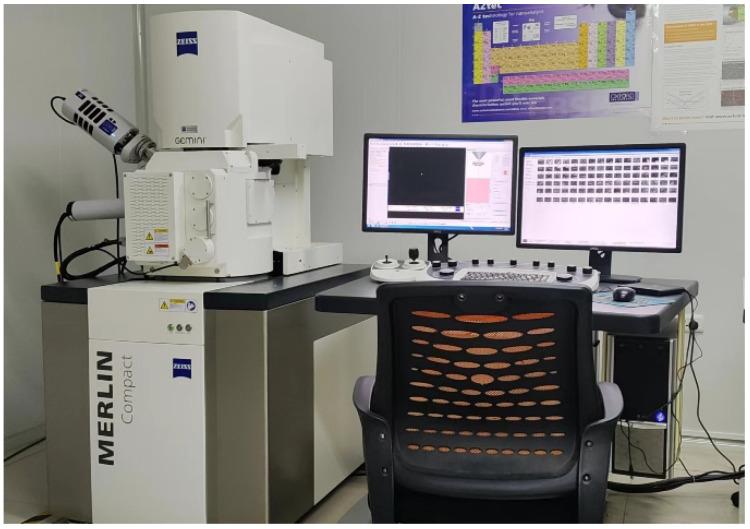
Scanning electron microscopy experiment.

The concrete samples used for the SEM experiment were crushed and sampled, and the central part of the concrete test block was taken for sample preparation. Use a cutting machine to cut small or sheet samples, making them have a relatively flat cross-section. Before the experiment, the samples need to be placed in an oven for drying, with the drying temperature maintained at 110 °C. Fix the dried samples to the test bench using conductive tape and spray gold onto the samples to make them conductive. The microstructure of the concrete samples was scanned using the scanning electron microscope produced by Zeiss of Germany, as shown in Figure 5. The test acceleration voltage is 5 kV, and the magnification is 5.00–10.00 k, which is used to observe the microscopic morphology and interface transition zone of UHPC.

### 2.5. MIP

**Figure 6:**
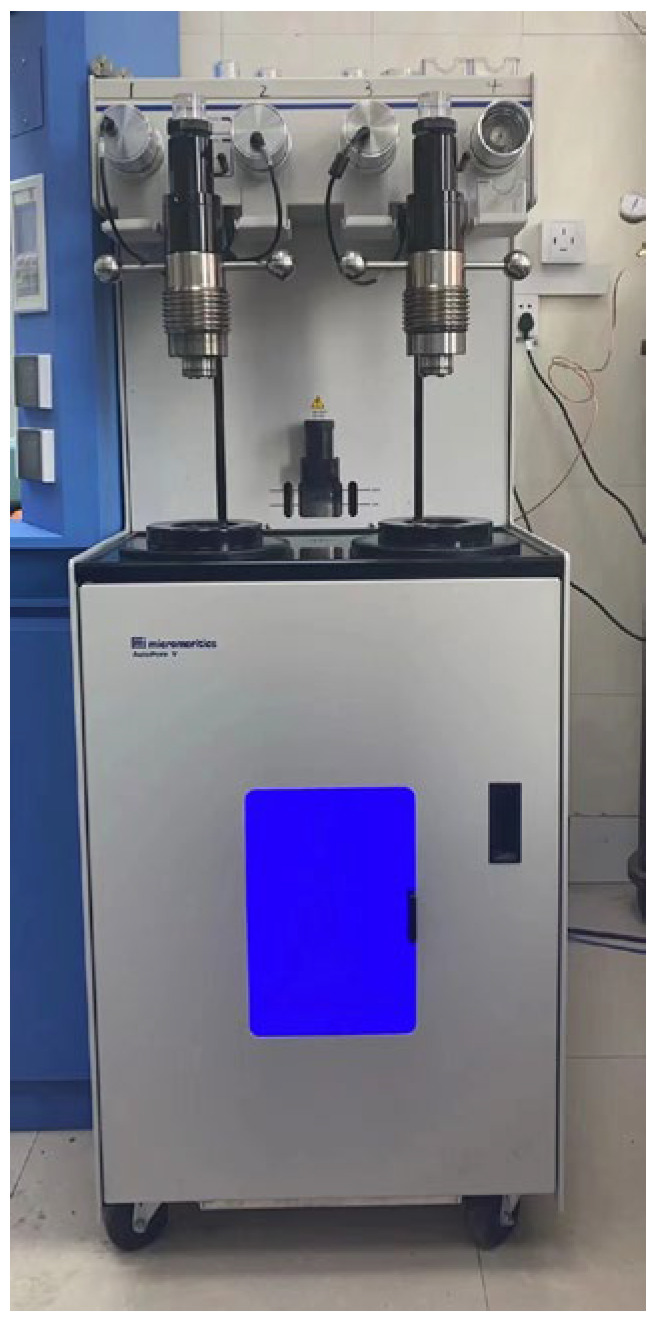
Mercury compression experiment.

The pore structure within the concrete was analyzed and detected using the mercury compression test. A total of six groups of test blocks, designated as S00, S10, S20, S30, S40, and S50, were selected. The samples were cut into small pieces with an edge length of about 1 cm by a cutting machine, dried, and then subjected to the mercury intrusion test. The experiment utilized the AutoPore V 9600 high-performance, fully automatic mercury porosimeter, produced by Micromeritics Instrument Corporation in Norcross, GA, USA, as shown in Figure 6. The pressurization range of the instrument is 0.10 to 61,000 psia, the surface tension of mercury is 0.485 N/m, and the contact angle θ is 130°.

## 3. Test Results and Analysis

### 3.1. Compressive Strength

**Figure 7:**
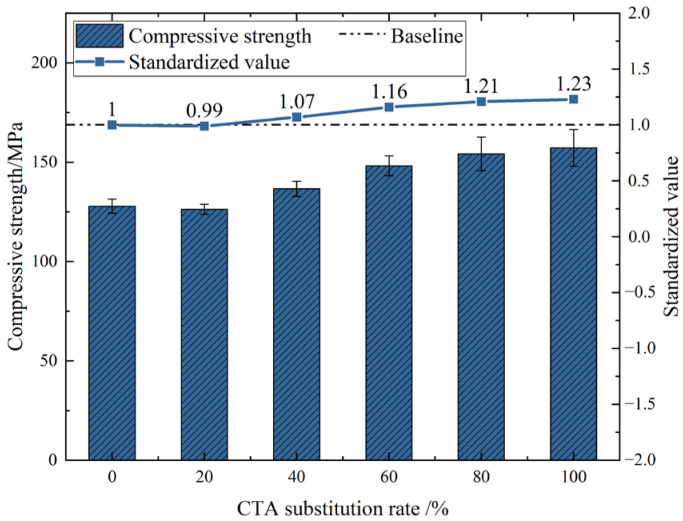
Compressive strength of UHPC with different CTA dosages.

The compressive strength of UHPC with different tile particle dosages after 28 days of curing is shown in Figure 7. It can be seen from the test results that when the substitution rates of CTA are 20%, 40%, 60%, 80%, and 100%, the compressive strengths increase by −1%, 7%, 16%, 21%, and 23%, respectively, compared with 0%. Overall, the addition of waste tile particles has improved the mechanical properties of UHPC to varying degrees. Among them, the tile particles with a 100% addition amount have the most significant increase in compressive strength, reaching 157.2 MPa. Roig-Floresd et al. [25] used CSCA (ceramic stoneware coarse aggregates) instead of natural gravel aggregate for compressive testing, and the results obtained showed that the compressive strength reached its maximum value at 100% CSCA, which is consistent with the experimental results in this paper and proves the positive effect on compressive strength.

The main reasons for this analysis are as follows. (1) The aggregate particles of ceramic tiles are rough and irregular in shape, mostly irregularly shaped particles with sharp edges and corners. When the mix ratio is the same, the amount of cement slurry coating increases, which is conducive to interface bonding and enhances the strength of concrete. (2) The ceramic tiles are made of siliceous aggregates. The calcium silicate hydrate generated by the reaction reduces the content of calcium hydroxide in the transition zone, thereby enhancing the strength of the interface transition zone between the ceramic tiles and the cement. (3) The uniformly distributed tile aggregates within the concrete, due to their relatively low apparent density and high water absorption rate, absorb a portion of water during the concrete preparation process. During the cement hydration process, the moisture inside the tiles transfers to the capillary pores of the cement, providing raw materials for the cement hydration reaction, enhancing the strength of the cement, and playing an internal curing role.

**Figure 8:**
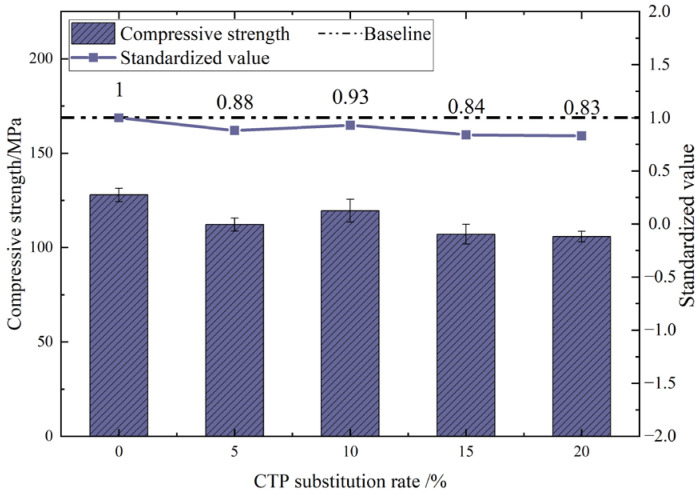
Compressive strength of UHPC with different CTP dosages.

The compressive strength of UHPC with different tile powder dosages after 28 days of curing is shown in Figure 8. When the substitution rates of CTP were 5%, 10%, 15%, and 20%, the compressive strengths decreased by 12%, 7%, 16%, and 17%, respectively, compared with 0%. The compressive strength of UHPC with added tile powder generally exhibits a trend of first decreasing, then increasing and then decreasing again with the increase in the amount of tile powder added. Overall, the addition of tile powder has a negative impact on the mechanical properties of UHPC, and this impact increases with the increase in the dosage. Among them, within the range of the test dosage, when the dosage is 10%, the influence on its compressive strength is the least, and the compressive strength value is 119.5 MPa. However, when the dosage is 20%, the decrease is relatively significant, and the compressive strength value is 105.8 MPa.

**Figure 9:**
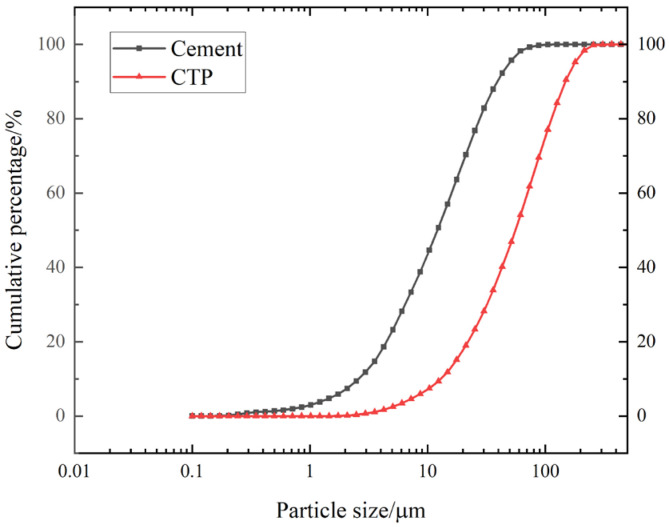
Particle size distribution of cement and CTP.

The main reasons for this analysis are as follows. (1) The addition of waste tile powder will produce a dilution effect. When using waste tile powder to replace part of the cement, the proportion of cement to hydration products will decrease accordingly. Especially when the dosage of waste tile powder is too high, the dilution effect becomes more pronounced. (2) As shown in Figure 9, the particle size of the tile powder used in the experiment was relatively large, which adversely affected the pozzolanic activity of the tile powder and reduced its pozzolanic activity. The role of tile powder in concrete is to serve as a filler, refining the internal structure of the concrete.

**Figure 10:**
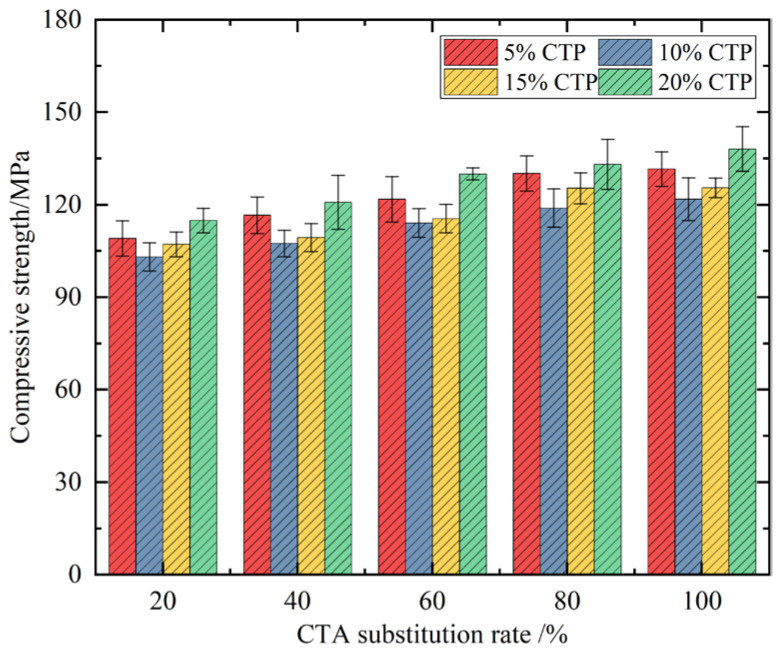
Compressive strength of UHPC in the case of double doping.

The compressive strength and its growth rate of UHPC after 28 days of curing under the condition of dual admixture are shown in Figure 10. According to the experimental results in the figure, the optimal dosage group of UHPC in the case of double addition of ceramic tile particles and ceramic tile powder is S54, which corresponds to 100% ceramic tile particle substitution and 20% ceramic tile powder substitution. This is mainly due to less effective participation in the pozzolanic reaction in the added CTP. However, during the production process of CTA, the surface of the CTA produced after being crushed by machines will be covered with a large amount of fine powder. The particle size of these powders is very small, which allows them to exert their pozzolanic reactivity fully. This part of the powder works together with the effective components in the previous CTP, thereby improving the mechanical properties of the concrete.

At 10% and 15% CTP content, the strength of concrete will be lower than 5% and 20% CTP content. At this time, the replacement amount of cement is obvious, so the dilution effect of the cement is greater than 5%, which will significantly weaken the strength of the concrete. When the content of CTP in concrete is further increased, the content of CTP that can play the role of pozzolana will increase, which will enhance the strength of concrete, so the strength of concrete will increase. In addition, when the content of CTA is small, the compressive strength of some concrete is lower than that of concrete without CTA and CTP. When the content of CTA is low, CTP has a greater effect on reducing concrete strength. Therefore, the strength of some concrete will be lower than that of the concrete without admixture.

### 3.2. Flexural Strength

**Figure 11:**
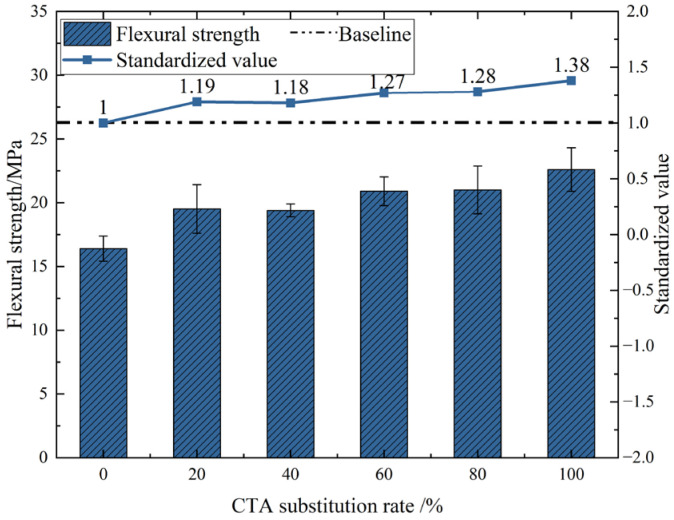
Flexural strength of UHPC with different CTA dosages.

The test prepared UHPC with different tile particle dosages and conducted flexural tests after 28 days of curing. The test results are shown in Figure 11. It can be seen from the figure that when the substitution rates of CTA are 20%, 40%, 60%, 80%, and 100%, the flexural strength increases by 19%, 18%, 27%, 28%, and 38%, respectively, compared to 0%. With the rise in the proportion of tile particles, the flexural strength of UHPC gradually increases. When the proportion of tile particles reaches 100%, the flexural strength reaches the maximum value, which is 22.6 MPa.

Due to the multi-angled, irregular shape and rough surface of the coarse aggregates of discarded tiles, interlocking effects will occur among the particles. In addition, the bonding area between the coarse aggregates of waste tiles and the cement slurry is larger than that of natural aggregates. Therefore, the aggregates of waste tiles can increase the contact area with cement mortar and provide a stronger bonding force. Additionally, during the preparation process of ceramic tile particle aggregates, manual crushing, mechanical grinding, and other processing procedures result in a significant amount of ceramic tile powder adhering to the surface of the ceramic tile particles. These adhering micro-powders can play a certain filling role in the gaps between the concrete aggregates.

**Figure 12:**
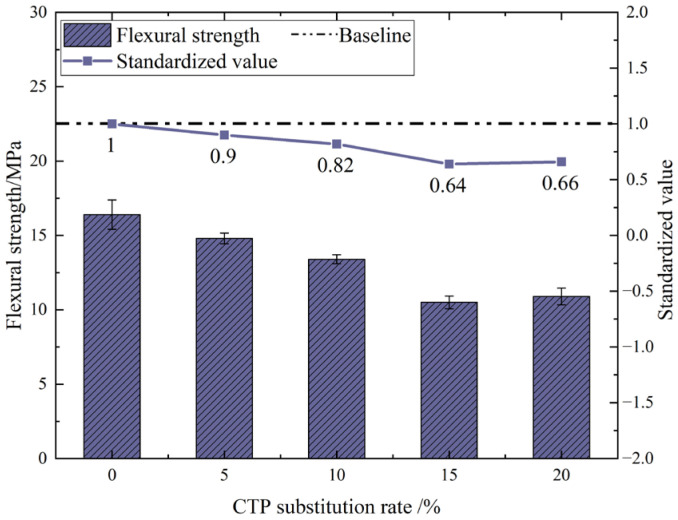
Flexural strength of UHPC with different CTP dosages.

Figure 12 shows the flexural strength values of UHPC after 28 days of curing when different dosages of tile powder were added to replace part of the cement. As shown in Figure 12, the addition of CTP results in a decrease in the flexural strength of UHPC to varying degrees. When the substitution rates of CTP were 5%, 10%, 15%, and 20%, the flexural strengths decreased by 10%, 18%, 36%, and 34%, respectively, compared with 0%. When the dosage was 15% in the test design, the decrease in flexural strength reached the maximum value, which was only 10.9 MPa.

Generally speaking, the UHPC matrix is a brittle material and is prone to brittle failure after bending, resulting in relatively poor bending resistance. UHPC enhances its toughness and flexural performance by adding steel fibers. The bonding strength between concrete and steel fibers is the primary factor influencing the flexural performance of concrete. The addition of waste tile powder will cause a dilution effect. As the amount of cement decreases, the ratio of cement to hydration products decreases accordingly. Especially when the amount of tile powder is too high, the dilution effect becomes more pronounced.

**Figure 13:**
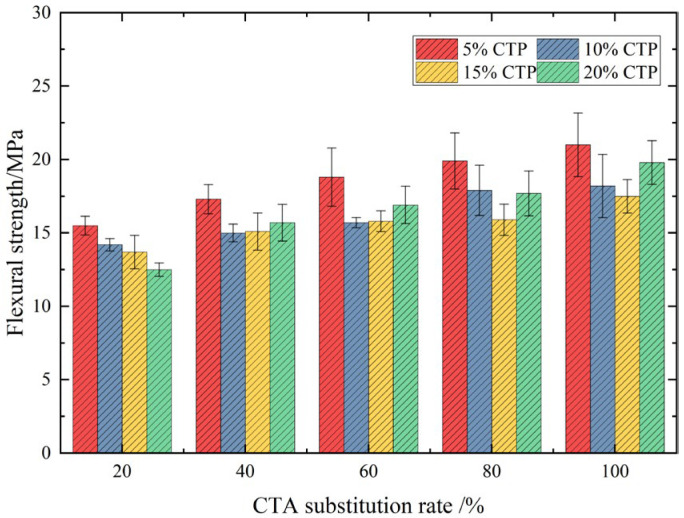
Flexural strength of UHPC in the case of double admixture.

Figure 13 shows the flexural strength of UHPC after 28 days of curing with double-mixed ceramic tile particles and ceramic tile powder. It can be observed from the experimental results in the figure that in the case of double admixture, its flexural strength has been improved to varying degrees compared to that of single tile powder admixture. The optimal dosage group for the flexural strength of UHPC in the case of double addition of ceramic tile particles and ceramic tile powder is S51, which corresponds to 100% ceramic tile particle substitution and 5% ceramic tile powder substitution.

### 3.3. Microstructure Characterization

**Figure 14:**
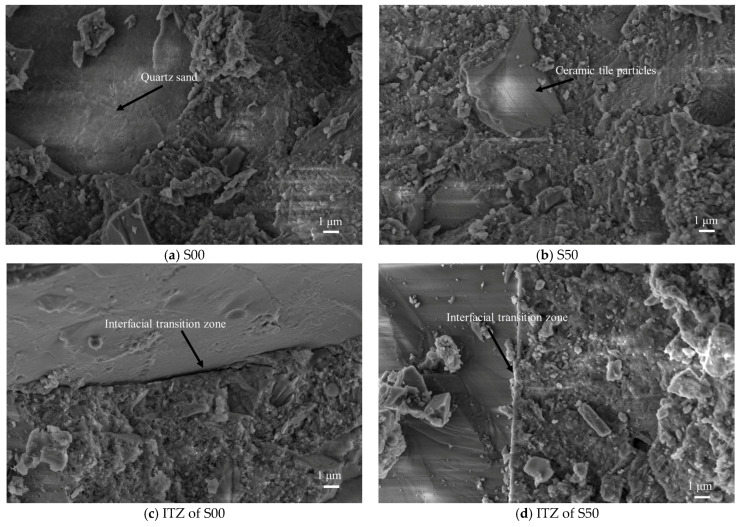
UHPC aggregates are combined with cement colloids.

The interfacial transition zone (ITZ), a structurally weak region in concrete attributed to the sidewall effect, critically influences the post-hardening performance of the material. Figure 14 illustrates the microstructural morphology of the aggregate–cement matrix interface in UHPC, as observed via SEM. As shown in Figure 14, after 28 days of curing, the quartz sand aggregates (Figure 14a) and tile particles (Figure 14b) become encapsulated within the cementitious matrix, forming a monolithic concrete structure. A discernible interface—identified as the ITZ—is observed between the cementitious binder and the aggregates.

Figure 14c and Figure 14d depict the microstructural characteristics corresponding to 0% and 100% CTA substitution, respectively. Comparative analysis reveals that the interfacial region between quartz sand and the cementitious matrix, under 0% CTA substitution, exhibits a greater presence of voids relative to the 100% CTA condition. At 100% CTA substitution, markedly denser interfacial bonding between CTA and the cementitious matrix is observed, accompanied by a significantly reduced ITZ width compared to the quartz sand–cement interface. Furthermore, the cementitious matrix surrounding CTA particles demonstrates a superior density compared to that surrounding conventional aggregates.

**Figure 15:**
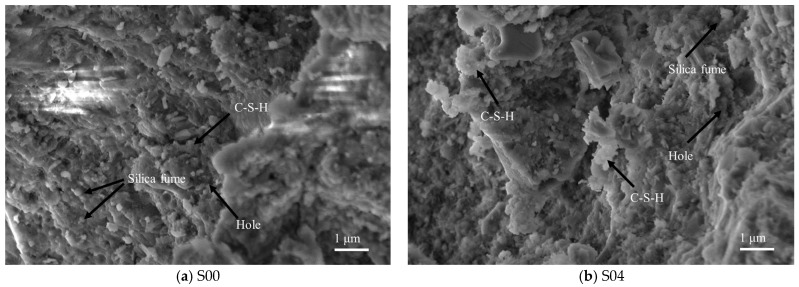
Microscopic morphology of UHPC.

During the hardening process of cement-based materials, the mixing water in the concrete mixture is consumed to participate in the hydration reaction. After the mixing water is consumed, pores will still be left inside the concrete. Moreover, polycarboxylate superplasticizers are ineffective in reducing these pores. It is necessary to rely on nano-scale active mineral admixtures to fill part of the pores caused by free water. As shown in Figure 15a,b, when using CTP to replace part of the cement in concrete preparation, the dosage of CTP increases, resulting in a continuous decrease in the amount of cement and a corresponding reduction in the ratio of cement to hydration products, which leads to an increase in porosity. However, the particle size of the added CTP is relatively large and cannot fill these pores effectively, which will cause the concrete's strength to decrease accordingly.

**Figure 16:**
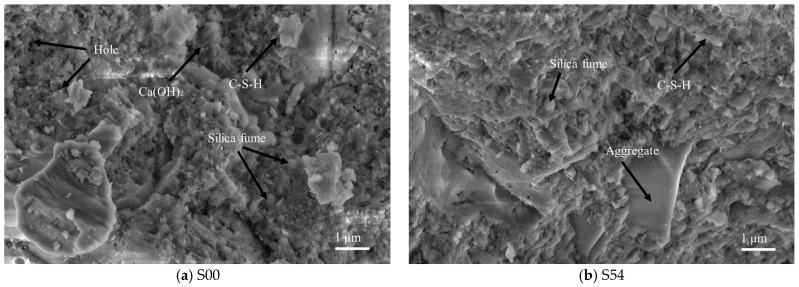
Hydration products of UHPC.

The calcium hydroxide generated by the hydration of cement exists in the form of a stable crystalline structure, which will compress the adhesion space of the C-S-H gel and inhibit the hydration reaction of cement to a certain extent. Because its strength is lower than that of the C-S-H gel, the calcium hydroxide aggregation area is relatively weak. When concrete is subjected to external loads, it begins to fail from its weak internal areas and then collapses as a whole. As shown in Figure 16a,b, the microstructure of the concrete without the addition of recycled tiles has a relatively large amount of weak calcium hydroxide, which is not conducive to the development of concrete strength. The microstructure of concrete mixed with recycled ceramic tiles is relatively dense, and the C-S-H gel produced by hydration develops well.

### 3.4. Pore Structure

**Figure 17:**
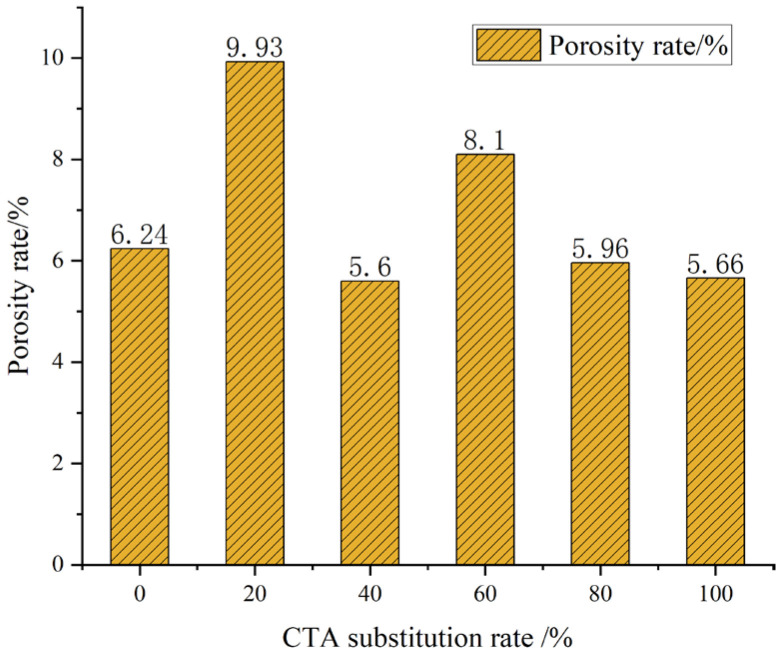
Porosity of UHPC single-doped ceramic particles.

As shown in Figure 17, the porosity of the concrete with CTA added ranges from 5.6% to 9.93%. Compared to the concrete without added CTA, the addition of CTA does not effectively reduce the porosity of the concrete. Instead, the porosity will increase at the addition amounts of 20% and 60%. The increase in concrete porosity will have a detrimental effect on the concrete’s strength. At the maximum porosity (20% CTA substitution), the enhancing effect of CTA on compressive strength was not reflected.

**Figure 18:**
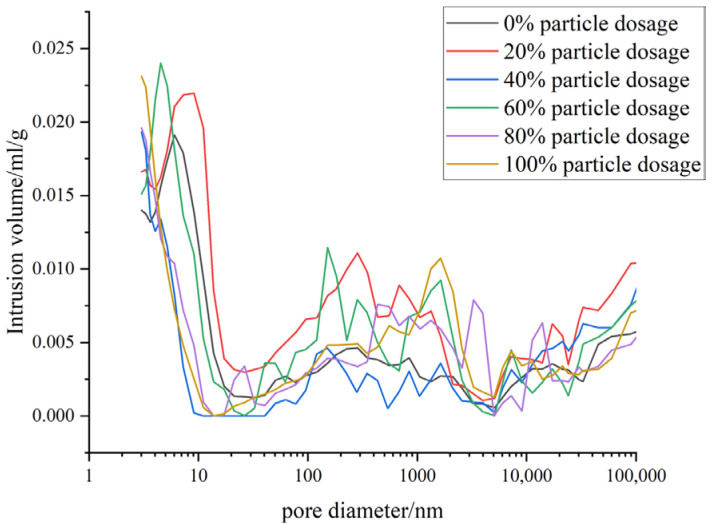
Pore size distribution of UHPC single-doped ceramic particles.

As illustrated in Figure 18, the primary pore diameter of concrete remains below 9.07 nm, and the incorporation of CTA significantly reduces the median pore size. There is a relatively high peak proportion in the pore size distribution range greater than 10,000 nm. These pores are primarily formed due to the introduction of gas during the preparation and mixing of concrete, which is difficult to discharge, resulting in large pores. In addition, the pore distribution in the mercury compression test includes the CTA. The pores of the CTA themselves are mainly capillary pores and macropores, which leads to a relatively high proportion of capillary pores and macropores in the concrete adulterated with CTA.

## 4. Conclusions

(1) The incorporation of CTP as a partial substitute adversely impacts the mechanical properties of ultra-high-performance concrete (UHPC). Both compressive and flexural strengths exhibited progressive deterioration with increasing CTP content. At a 20% substitution level, UHPC demonstrated 17% and 34% reductions in compressive and flexural strength, respectively.
(2) Incorporating CTA into UHPC progressively enhanced both compressive and flexural strengths with increasing CTA content. Peak strengths of 157.2 MPa (compressive) and 22.6 MPa (flexural) were achieved at 100% CTA replacement. The microstructure analysis of SEM and MIP showed that the substitution of CTA refined the ITZ and reduced the median pore size. These microstructural modifications collectively facilitated strength development in the concrete matrix.
(3) When CTP and CTA are added to UHPC at the same time, the weakening effect of CTP on the mechanical properties of UHPC still exists, and the incorporation of CTP will offset part of the enhancement effect of CTA on the mechanical properties of UHPC. In the case of double admixtures, the compressive strength is best when 100% CTA and 20% CTP are used, the microstructure of concrete is more compact than that of concrete without admixtures, and the flexural strength is best when 100% CTA and 5% CTP are used.
(4) In this paper, an experiment was designed to study the effect of waste ceramic tiles on the mechanical properties of UHPC. However, there are still some areas worth further study. In this experiment, only one mix proportion was considered, and the waste ceramic tiles have high water absorption. In future research, a higher water–binder ratio can be considered to study its mechanical properties. UHPC can also be prepared by using finer CTP powder or enhancing its pozzolanic activity through the use of an alkali activator. In addition, the test pieces in the test are made of raw materials from fixed manufacturers. Different suppliers may have varying material characteristics due to their unique manufacturing processes, which is also one reason for the possible deviation in test results.

## Figures and Tables

**Table 1:** Chemical composition of cement and CTP%.

	CaO	SiO_2_	AL_2_O_3_	Fe_2_O_3_	MgO	SO_3_	CL^−^	Loss
Cement	56.77	20.86	5.90	3.61	3.50	2.43	0.02	1.16
CTP	0.90	70.68	19.56	1.30	1.12	—	—	0.09

**Table 2:** Gradation of CTA.

Particle size/mm	2.36~1.18	1.18~0.6	0.6~0.3	0.3~0.15
Distribution rate/%	44	25.4	18	12.6

**Table 3:** Performance of CTA.

	Particle Size/mm	Water Content/%	Water Absorption Rate/%	Apparent Density (kg/m^3^)	Bulk Density (kg/m^3^)	Apparent Density (kg/m^3^)
CTA	0.15~2.36	0.51	16.22	2470	1420	2470

**Table 4:** UHPC mix proportion.

Cementitious Materials	Water	Aggregate	Water Reducing Agent (wt. %)	Steel Fiber (Vol. %)
1.0	0.18	1	1.5	2

**Table 5:** Dosage of UHPC basic materials.

Cement (kg/m^3^)	Silica Fume (kg/m^3^)	Water (kg/m^3^)	Fine Aggregate (kg/m^3^)	Water Reducing Agent (kg/m^3^)	Steel Fiber (kg/m^3^)
1035	115	207	1150	17.3	156

## Data Availability

The original contributions presented in this study are included in the article. Further inquiries can be directed to the corresponding authors.
